# Navigating change: the ongoing efforts to contain poliovirus in the United States

**DOI:** 10.3389/fbioe.2025.1756311

**Published:** 2026-01-15

**Authors:** Christy Ottendorfer, Lia Haynes Smith, Shelley Jorgensen

**Affiliations:** 1 U.S. Centers for Disease Control and Prevention, Office of Readiness and Response, Division of Regulatory Science and Compliance, U.S. National Authority for Containment of Poliovirus, Atlanta, GA, United States; 2 U.S. Centers for Disease Control and Prevention, Office of Readiness and Response, Division of Regulatory Science and Compliance, Atlanta, GA, United States

**Keywords:** biosafety, poliovirus, WHO, laboratory biorisk management, USA

## Abstract

United States (U.S.) poliovirus research and vaccination programs have eliminated polio in the U.S. and brought the world to the brink of polio eradication. As part of a global action plan, the U.S. committed to safeguard the polio eradication efforts through identification of domestic facilities holding poliovirus materials and implementing stringent laboratory biocontainment measures overseen by U.S. CDC. In January 2025, the White House announced the withdrawal of the U.S. from the WHO. Consistent with the administration’s policy and guidance, the U.S. CDC is developing a new framework for poliovirus containment independent of WHO to protect the health security of the U.S. from the continued threat of poliovirus and ensure the long-term success of polio eradication.

## Introduction

**FIGURE 1:**
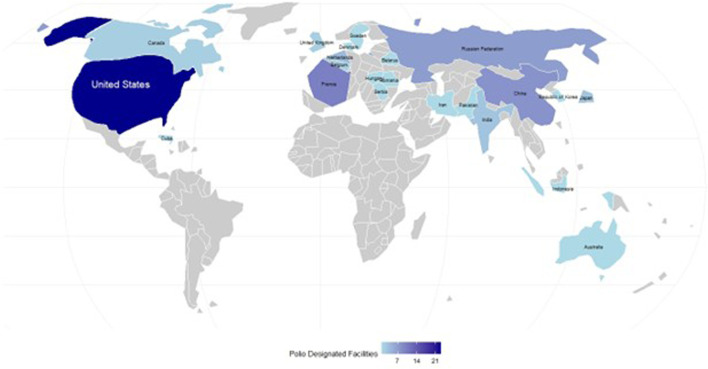
Cartogram of countries hosting poliovirus designated facilities, April 2025. Facilities designated to continue to use or store polioviruses post-eradication (n–76) were distributed in 23 countries, with countries resized based on number of reported designated facilities. The largest number of poliovirus laboratories were located in the United States. Countries shaded gray did not plan to retain poliovirus materials post-eradication. Data source: World Health Organization (2025).

Poliomyelitis (polio) is a highly infectious viral disease that largely affects children with as many as 1 in 200 infections leading to irreversible paralysis (Centers for Disease Control and Prevention (CDC), 2024). There is no cure for polio; it can only be prevented through vaccination (Centers for Disease Control and Prevention (CDC), 2024). Although eliminated from the United States (U.S.) in 1979 (Strebel et al., 1992), a resurgence in poliovirus has been detected in several countries (Molodecky et al., 2025) and continues to pose a national security risk whether through importation (Link-Gelles et al., 2022) or a breach in laboratory containment (Bandyopadhyay et al., 2019). The U.S. also has the largest number of designated poliovirus laboratory facilities in the world (Figure 1) (World Health Organization, 2025) but a continued decline in national childhood vaccination coverage (Seither et al., 2024) presents a potentially dynamic threat requiring effective containment strategies to mitigate accidental facility-associated reintroduction of the virus into communities. In other countries, routine wastewater monitoring of poliovirus laboratories has occasionally discovered environmental contamination and asymptomatic infection of laboratory workers from facility accidents-important findings that have been used to inform emergency response plans and enhance laboratory biocontainment practices by national authorities and poliovirus laboratories (Duizer et al., 2023). Areas with low vaccination rates are especially concerning, as a single case of paralytic polio, such as the one reported in New York State in 2022 (Link-Gelles et al., 2022), requires an enhanced public health response. This situation not only has economic implications but also necessitates ongoing vigilance from polio-free countries until global eradication is achieved (Huseynov et al., 2025).

The U.S. pioneered research and vaccine development to protect children against poliomyelitis resulting in the first licensed inactivated polio vaccine in 1955 by Jonas Salk, followed by a licensed attenuated oral polio vaccine in the 1960s by Albert Sabin, and a new oral polio vaccine with improved genetic stability introduced in 2021 by a public-private partnership (Lopez Cavestany et al., 2024). Polio eradication efforts have eliminated wild poliovirus transmission from all but two countries (Lopez Cavestany et al., 2024); however, laboratories still need poliovirus to produce life-saving vaccines, validate diagnostic tests to detect polio if it reemerges, study antiviral drugs in case of a poliovirus outbreak or bioterrorism event, monitor for silent transmission through environmental sampling, and maintain rapid response capability to detect and control reintroduction of poliovirus from domestic incidents or importation (World Health Organization, 2025; Lopez Cavestany et al., 2024). It is up to the U.S. to ensure that domestic laboratories handling poliovirus meet strict containment and biosafety standards to protect public health and prevent accidental outbreaks in our communities.

## Main text

As part of a global action plan, the U.S. committed to safeguard the polio eradication efforts through identification of domestic facilities holding poliovirus materials (via a national survey) and implementation of stringent laboratory biocontainment measures (World Health Organization, 2018) overseen by a national authority for containment (U.S. NAC) at the U.S. Centers for Disease Control and Prevention (U.S. CDC) (Ottendorfer et al., 2024). The U.S. CDC protects the nation from preventable poliovirus-related threats. The U.S. NAC ensures that poliovirus materials collected, tested, destroyed, or transferred by domestic facilities are handled in a safe and secure manner. It audits and certifies facilities handling poliovirus materials to ensure compliance with containment safeguards to protect communities. The U.S. NAC’s activities are important to the CDC mission to increase the health security of America by ensuring critical scientific research in poliovirus laboratories is conducted as safely and securely as possible. This research leads to discoveries that can save lives and help protect the American people’s health. The U.S. NAC leads efforts to strengthen national biocontainment oversight using industry-leading laboratory biorisk and quality management system standards, provides technical guidance on laboratory biorisk management system implementation, responds to reported laboratory incidents, and conducts research for continual improvement of laboratory biosafety. The U.S. NAC’s activities have led to improved biocontainment practices for the use and storage of poliovirus, facilitating a 67% reduction in designated poliovirus facilities in the United States, as many opted to instead destroy, inactivate, or transfer infectious materials (Ottendorfer et al., 2024). The U.S. NAC effectively established and continues to maintain a national inventory and implementation of robust biosafety and biosecurity requirements through partner (e.g., facilities and state partners) collaboration and engagement.

In January 2025, the White House announced Executive Order (EO) 14155, titled “Withdrawing the United States from the World Health Organization” (WHO) (United States Government White House, 2025). Consistent with the administration’s policy and guidance, the U.S. NAC is developing a new framework for certification to become a global leader in poliovirus containment. The U.S. NAC previously certified poliovirus designated facilities using the WHO containment certification scheme, coordinated external containment training with WHO, and shared annual reports and certification applications with the Global Certification Commission for poliomyelitis eradication (an independent body reporting to the WHO Director General to oversee containment and certify the world is polio-free) (World Health Organization, 2025). Going forward, the U.S. NAC is reviewing the national containment certification process and considering other standards, such as the International Organization for Standardization (ISO) 17021-1:2015 and 35001:2019 standards, relevant national guidelines, and technical guidance to bolster U.S. containment efforts. Development, clearance, and publication of the framework are anticipated within the next 6 months. Further, U.S. NAC will use a national certification committee and collaborate with federal partners to support implementation and independent assurance of containment best practices within the United States.

## Discussion

Poliovirus containment programs are led by national authorities in 20+ countries to mitigate any risks that may be posed by facilities that conduct essential vaccine research, production, and surveillance (World Health Organization, 2025). The U.S. NAC also collaborates with other country NACs and helped establish an independent technical group focused on poliovirus containment (i.e., International NAC or iNAC). This group operates independently from the WHO and is responsible for harmonizing policies and guidelines for effective implementation of poliovirus containment measures. The U.S. will continue bilateral engagements with other countries and technical consultation with the iNAC to aid in protecting public health through certification of facilities designated to retain this high-consequence pathogen. U.S. CDC will continue to safeguard public health through immunization programs, poliovirus outbreak simulation exercises to test and refine the national poliovirus response plan, inventory poliovirus materials using a national poliovirus containment survey, and oversight of domestic facilities retaining polioviruses consistent with national and international biorisk management system standards, independent from WHO. These efforts are essential to protecting the health security of the U.S. from the continued threat of poliovirus and ensuring the long-term success of polio eradication.

## Data Availability

The original contributions presented in the study are included in the article/supplementary material, further inquiries can be directed to the corresponding author.
